# Tau and neurodegeneration

**DOI:** 10.1002/cm.21812

**Published:** 2023-12-10

**Authors:** Michel Goedert, R. Anthony Crowther, Sjors H. W. Scheres, Maria Grazia Spillantini

**Affiliations:** 1 Medical Research Council Laboratory of Molecular Biology, Francis Crick Avenue, Cambridge, UK; 2 Department of Clinical Neurosciences, University of Cambridge, Cambridge, UK

**Keywords:** tau filaments, tau gene mutations, tau isoforms, tauopathies

## Abstract

First identified in 1975, tau was implicated in Alzheimer’s disease 10 years later. Filamentous tangle inclusions were known to be made of hyperphosphorylated tau by 1991, with similar inclusions gaining recognition for being associated with other neurodegenerative diseases. In 1998, mutations in *MAPT*, the gene that encodes tau, were identified as the cause of a dominantly inherited form of frontotemporal dementia with abundant filamentous tau inclusions. While this result indicated that assembly of tau into aberrant filaments is sufficient to drive neurodegeneration and dementia, most cases of tauopathy are sporadic. More recent work in experimental systems showed that filamentous assemblies of tau may first form in one brain area, and then spread to others in a prion-like fashion. Beginning in 2017, work on human brains using high-resolution techniques has led to a structure-based classification of tauopathies, which has opened the door to a better understanding of the significance of tau filament formation.

## 1 Introduction

Publication of this article marks the 50th anniversary of the description and naming of tau (Tubulin-Associated Unit) by Weingarten et al. (1975). At the time of its discovery, one could not have predicted that interest in tau would increase so dramatically; this happened because of the finding that tau is the main component of the filamentous inclusions that characterize many human neurodegenerative diseases that have been dubbed tauopathies. The most common of these diseases is Alzheimer’s disease (AD), with others including chronic traumatic encephalopathy (CTE), Pick’s disease (PiD), progressive supranuclear palsy (PSP), globular glial tauopathy (GGT), corticobasal degeneration (CBD), and argyrophilic grain disease (AGD). A great deal of work was needed between 1985 and 1991 to firmly establish that tau is the main component of the aberrant filamentous tangles of AD (Brion et al., 1985; Delacourte et al., 1986; Goedert et al., 1988; Grundke-Iqbal, Iqbal, Quinlan, et al., 1986; Grundke-Iqbal, Iqbal, Tung, et al., 1986; Kondo et al., 1988; Kosik et al., 1986, 1988; Lee et al., 1991; Nukina & Ihara, 1986; Wischik, Novak, Edwards, et al., 1988; Wischik, Novak, Thøgersen, et al., 1988; Wood et al., 1986).

Tangle pathology was identified by Alois Alzheimer via light microscopy (Alzheimer, 1907), after which Michael Kidd used electron microscopy to show that the tangles are composed of abnormal filaments, which he named paired helical filaments (PHFs; Kidd, 1963). Identified as minority species were straight filaments (SFs), which do not exhibit the modulation in width shown by PHFs. Tau was first reported to have a role in neurodegeneration by Brion et al., who showed labelling of the tangle pathology of AD by a tau-specific antibody (Brion et al., 1985).

## 2 Tau Isoforms

Martin Roth brought the tangle problem to the “MRC Laboratory of Molecular Biology” where, under the direction of Aaron Klug, biochemical, immunological, structural, and molecular biological techniques were used, to find out what PHFs are made of. In conducting this work, we also characterized human tau isoforms.

Since the PHF is defined by its ultrastructure, electron microscopy alone is insufficient to reveal its component parts. Required was a label for individual filaments using microscopy, as well as for the protein bands revealed by gel electrophoresis from successively purified tangle preparations. The protein bands could then be partially sequenced, so that cDNA clones could be isolated and sequenced.

Claude Wischik and Tony Crowther used proteases to break down the insoluble tangles, in order to study the structural organization of PHFs (Crowther & Wischik, 1985; Wischik et al., 1985). To obtain a label for purified PHF components, Michal Novak and Cesar Milstein produced monoclonal antibodies, one of which (6–423) decorated individual PHFs isolated from tangle fragments in electron microscopy and also labelled a 12 kDa protein gel band extracted from purified PHF preparations. The partial amino acid sequence of this band was then determined by John Walker, after which Michel Goedert isolated and sequenced cDNAs from a human brain library. A striking repeat pattern was revealed by the deduced amino acid sequences that was unrelated to any known sequence. A major 6 kb and a minor 2 kb band were observed by RNA blotting, and these were identical to the pattern obtained using a cDNA clone encoding murine tau (Drubin et al., 1984), kindly provided by Gloria Lee and Marc Kirschner.

Lee and Kirschner had deduced the amino acid sequence of a form of murine tau after sequencing this and other cDNA clones; when Klug read part of our sequence over the telephone to Kirschner, it was clear that we had established tau as an integral component of PHFs (Goedert et al., 1988; Wischik, Novak, Edwards, et al., 1988; Wischik, Novak, Thøgersen, et al., 1988). Following this work, studies using electron microscopy and image reconstruction demonstrated that PHFs and SFs are each composed of two identical C-shaped subunits of tau that are linked differently, giving rise to their characteristic morphologies (Crowther, 1991).

**Figure 1:**
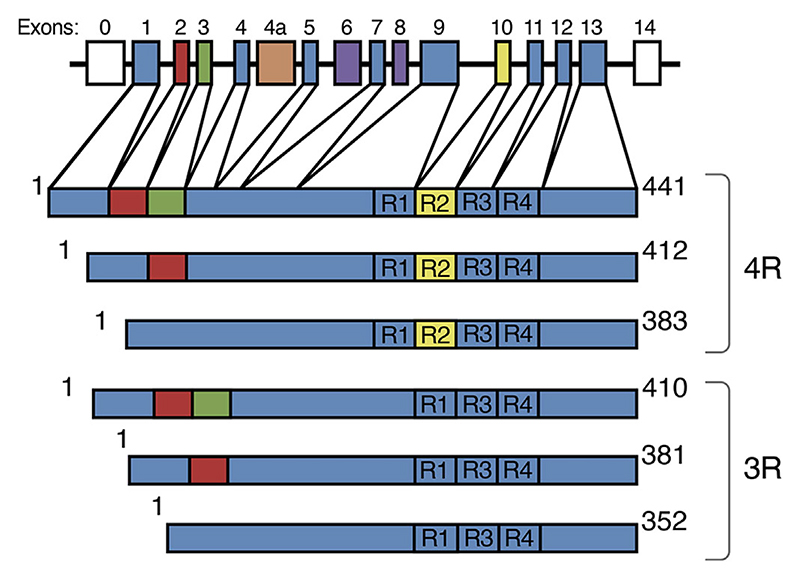
Human brain tau isoforms. *MAPT* and the six tau isoforms expressed in adult human brains. *MAPT* consists of 14 exons (E). Alternative mRNA splicing of E2 (red), E3 (green) and E10 (yellow) gives rise to six tau isoforms (352–441 amino acids). The constitutively spliced exons (E1, E4, E5, E7, E9, E11, E12, and E13) are shown in blue. E6 and E8 (violet) are not transcribed in human brains. E4a (orange) is only expressed in the peripheral nervous system. The repeats (R1-R4) are shown, with three isoforms having four repeats (4R) and the other three isoforms having three repeats (3R).

January 1988 saw reporting of the first sequence of an isoform of murine tau with three repeats in the microtubule-binding region (0N3R; Lee et al., 1988), followed by that of the sequence of an isoform of human tau (0N3R) in June of that year (Goedert et al., 1988). In the following year, we identified a four-repeat isoform of human tau (0N4R; Goedert, Spillantini, Potier, et al., 1989), as well as the tau isoforms that are expressed in adult human brains (0N3R, 1N3R, 2N3R, 0N4R, 1N4R, and 2N4R, Figure 1; Goedert, Spillantini, Jakes, et al., 1989). Six tau isoforms expressed by alternative mRNA splicing in adult bovine brains were described in parallel studies (Himmler, 1989; Himmler et al., 1989). The expression of human tau isoforms from their cDNAs and alignment with dephosphorylated adult brain tau established the identification by our group of the major tau isoforms (Goedert & Jakes, 1990). Two years later, big tau, an isoform that nearly doubles the molecular mass of the brain isoforms, was described as the predominant tau isoform in the peripheral nervous system (Couchie et al., 1992; Goedert, Spillantini, & Crowther, 1992).

The presence or absence of three inserts defines the six isoforms of brain tau that range from 352 to 441 amino acids, and these are generated through alternative mRNA splicing of the *MAPT* gene (Figure 1). Inserts of 29 or 58 amino acids (1N and 2N) are located near the N-terminus and one insert is in the C-terminal half. The latter consists of repeat R2 in the three isoforms with four repeats (4R), whereas the other three isoforms have three repeats (3R). Exons 9–12 of *MAPT* encode repeats 1–4 (Figure 1). 3R and 4R tau are present at similar levels in adult human brains (Goedert & Jakes, 1990). Only the shortest tau isoform (0N3R) is present in developing human brains. Together with some adjoining sequences, the repeats constitute the microtubule-binding domains of tau (reviewed in Wang & Mandelkow, 2015), and also the cores of filamentous tau in neurodegenerative diseases; this suggests that physiological function and pathological assembly are mutually exclusive. Although often said to stabilize axonal microtubules and promote their assembly, tau is in fact enriched in their more labile domains, where it promotes assembly (Qiang et al., 2018).

The isoform composition of tau is not conserved across species. 3R, 4R, and 5R tau isoforms are expressed in adult chicken brains (Yoshida & Goedert, 2002), while most adult rodents express only 4R tau (Götz et al., 1995). The *Caenorhabditis elegans* and *Drosophila melanogaster* genomes each encode one protein with tau-like repeats (Goedert, Baur, et al., 1996; Heidary & Fortini, 2001). The high-molecular weight proteins MAP2 and MAP4 display similar repeats (Aizawa et al., 1990; Lewis et al., 1988). MAP2 and tau likely shared a recent common ancestor, whereas MAP4 probably derives from a nonvertebrate ancestor (Sündermann et al., 2016).

## 3 Tau Assemblies

Tau research was made difficult for many years by the insolubility of PHFs in tangle fragments. A method based on sarkosyl solubility (Greenberg & Davies, 1990), which enriches for less insoluble PHFs, mainly made of full-length tau, was a pivotal step forward. Antibodies specific for the N- and C-termini of tau decorated these filaments in negative stain immunoelectron microscopy (Goedert, Spillantini, Cairns, & Crowther, 1992), but this was not the case for antibodies against repeats R3 or R4 of tau, because their epitopes are occluded in the filaments. This work, together with the results of biochemical studies, supported the view that tau filaments are made of an ordered core and a protease-sensitive fuzzy coat (this distinction was first made by Wischik, Novak, Edwards, et al., 1988). The core is required for a filament to look like a filament, whereas the fuzzy coat makes up the rest of the tau molecule. Tau filaments have the biophysical characteristics of amyloid (Berriman et al., 2003).

Tau’s ability to interact with microtubules is negatively regulated by phosphorylation (Lindwall & Cole, 1984) and filamentous tau is aberrantly hyperphosphorylated (Grundke-Iqbal, Iqbal, Tung, et al., 1986). It is unknown if phosphorylation is necessary and/or sufficient for the assembly of tau into filaments in the brain. In addition to phosphorylation, filamentous tau is also known to undergo acetylation, glycation, isomerization, O-GlcNAcylation, nitration, sumoylation, ubiquitination, and truncation (reviewed in Goedert et al., 2017). Acetylation of lysine residues (21 of which are located between residues 244 and 380) reduces charge, which may play a role in the filament assembly of tau.

All six tau isoforms are present in disease filaments in AD, CTE, familial British dementia (FBD), familial Danish dementia (FDD), primary age-related tauopathy (PART), and some other diseases, whereas Pick bodies are only made of 3R tau. 4R tau makes up the filaments in CBD, AGD, PSP, and GGT. Tau filament morphologies vary in different diseases, even when comprised of the same isoforms (reviewed in Goedert et al., 2017).

Attempts to assemble recombinant tau into filaments began in the early 1990s. This was possible with a fragment containing the repeat region (Crowther et al., 1992; Wille et al., 1992), but full-length tau resisted assembly. Only when negatively charged substances, such as sulfated glycosaminoglycans, were used, did full-length tau also assemble into filaments (Goedert, Jakes, et al., 1996; Pérez et al., 1996). These filaments were decorated by antibodies against the N- and C-termini of tau, but not by an antibody specific for the repeats.

In vitro (von Bergen et al., 2000), in cells (Falcon et al., 2015), and in transgenic mice (Macdonald et al., 2019), a hexapeptide sequence in R3 (VQIVYK, amino acids 306–311) is needed for filament assembly. Steric zippers are formed by microcrystals of residues 306–311(Sawaya et al., 2007). Residues 310–313 in tau (YKPV) differ from the equivalent residues in MAP2 (TKKI). When the latter were changed to YKPV, MAP2c also assembled (Xie et al., 2015).

## 4 Tau Genetics

Human genetics established the link between tau dysfunction and neurodegeneration. A dominantly inherited form of frontotemporal dementia and parkinsonism was found to be associated with chromosome 17q21–22 (Wilhelmsen et al., 1994), the region where *MAPT* resides (Neve et al., 1986). Then, in June 1998, mutations in *MAPT* were linked to a type of frontotemporal dementia associated with parkinsonism (Hutton et al., 1998; Poorkaj et al., 1998; Spillantini et al., 1998). Filamentous inclusions composed of 3R, 4R, or 3R + 4R tau were found in neurons or in both neurons and glia (reviewed in Goedert et al., 2017). Deposits of Aβ outside of cells, a defining characteristic of AD, were not present.

**Figure 2:**
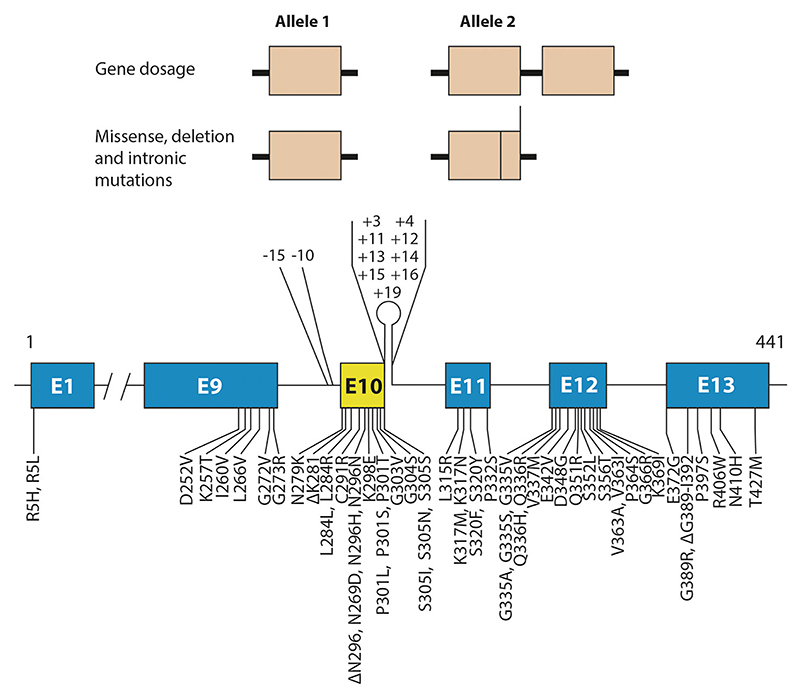
Mutations in *MAPT* in FTDP-17 T. Gene dosage mutations, where one allele of *MAPT* is doubled, as well as missense, deletion and intronic mutations in *MAPT* are dominantly inherited. (Fifty-five coding region and eleven intronic mutations are shown.) They give rise to 65 different forms of FTDP-17 T (pathogenic intronic mutation –15/+4 is compound heterozygous).

Now, it was known that a mechanism going from monomeric to filamentous tau is sufficient to cause neurodegeneration and associated cognitive deficits. By December 2023, 65 pathogenic *MAPT* mutations had been identified (Figure 2). Behavioral symptoms are the most common clinical manifestation, but in some cases, *MAPT* mutations are also associated with parkinsonism. Neurological syndromes like those of PiD, PSP, GGT, CBD, and motor neuron disease have been described. Onset of disease symptoms is variable but can be as early as in the third decade.

Most *MAPT* mutations are concentrated in exons 9–12 (encoding repeats R1–R4) and the introns flanking exon 10, with a smaller number in exon 13. Exon 1 is where the R5H and R5L mutations are located. Gene dosage mutations can also give rise to FTDP-17T, indicating that the overexpression of wild-type tau is sufficient to cause disease (Wallon et al., 2021).

Some mutations have a primary effect at the protein level, whereas others impact the alternative splicing of tau pre-mRNA. The latter can be intronic or exonic, with 3R or 4R tau isoforms being overexpressed and assembling into disease filaments. These findings indicate that the healthy ratio of 3R:4R tau in adult human brains is pivotal for preventing neurodegeneration and dementia.

Pathogenic mutations in *MAPT* led to the production of transgenic rodent lines that show neurodegeneration and abundant tau filaments (Allen et al., 2002; Götz et al., 2001; Lewis et al., 2000; Yoshiyama et al., 2007). Tau aggregation correlates with neurodegeneration (Macdonald et al., 2019).

Transgenic mouse lines led in turn to the identification of the prion-like properties of assembled tau (Clavaguera et al., 2009), which parallel the staging of tau pathology in AD (Braak & Braak, 1991). Intracerebral injection of brain extracts from a mouse line transgenic for human mutant tau with abundant tau inclusions into a line transgenic for wild-type human tau without inclusions led to the assembly of wild-type human tau and the propagation over time of inclusions to distant brain areas. Short filaments from brain extracts of mice transgenic for human P301S tau were then shown to have the greatest seeding activity (Jackson et al., 2016). Producing “tauopathy in a dish” is an ongoing goal, with work on induced pluripotent stem cell-derived cortical neurons from patients with *MAPT* splicing mutation N279K showing earlier expression of 4R tau than controls (Iovino et al., 2015).

## 5 Tau Filament Structures From Human Brains

The high-resolution structures of tau inclusions remained unknown, even decades after the presence of inclusions had been shown in human brains. This changed in 2017, when electron cryo-microscopy (cryo-EM) made it possible (He & Scheres, 2017) to determine the structures of amyloid filaments from human brains (Fitzpatrick et al., 2017).

Cryo-EM structures of PHFs and SFs that were extracted from the frontal cortex of an individual with AD showed that each filament type comprises two identical protofilaments with a C-shaped ordered core, the Alzheimer tau fold (Fitzpatrick et al., 2017). PHFs and SFs are distinguished by different packings of protofilaments. The ordered core, which forms a β-sheet-rich structure that is characteristic of amyloids, comprises amino acids 306–378 (in the numbering of the 441 amino acid tau isoform). The tau filament core thus consists of the whole of R3 and R4, and 10–13 amino acids after R4. Tau monomers can be incorporated into the filaments, regardless of whether they contain R2, accounting for the presence of all six tau isoforms in PHFs and SFs in AD (Fitzpatrick et al., 2017; Goedert, Spillantini, Cairns, & Crowther, 1992). The remaining 80% of tau forms the fuzzy coat. Other AD cases displayed the same tau filament structures in frontal cortex (Falcon, Zhang, Schweighauser, et al., 2018), and this was true also of tau filaments from different brain regions of individuals with AD. Unknown is what connections may exist between intracellular inclusions of tau and extracellular deposits of Aβ (Bloom, 2014).

**Figure 3:**
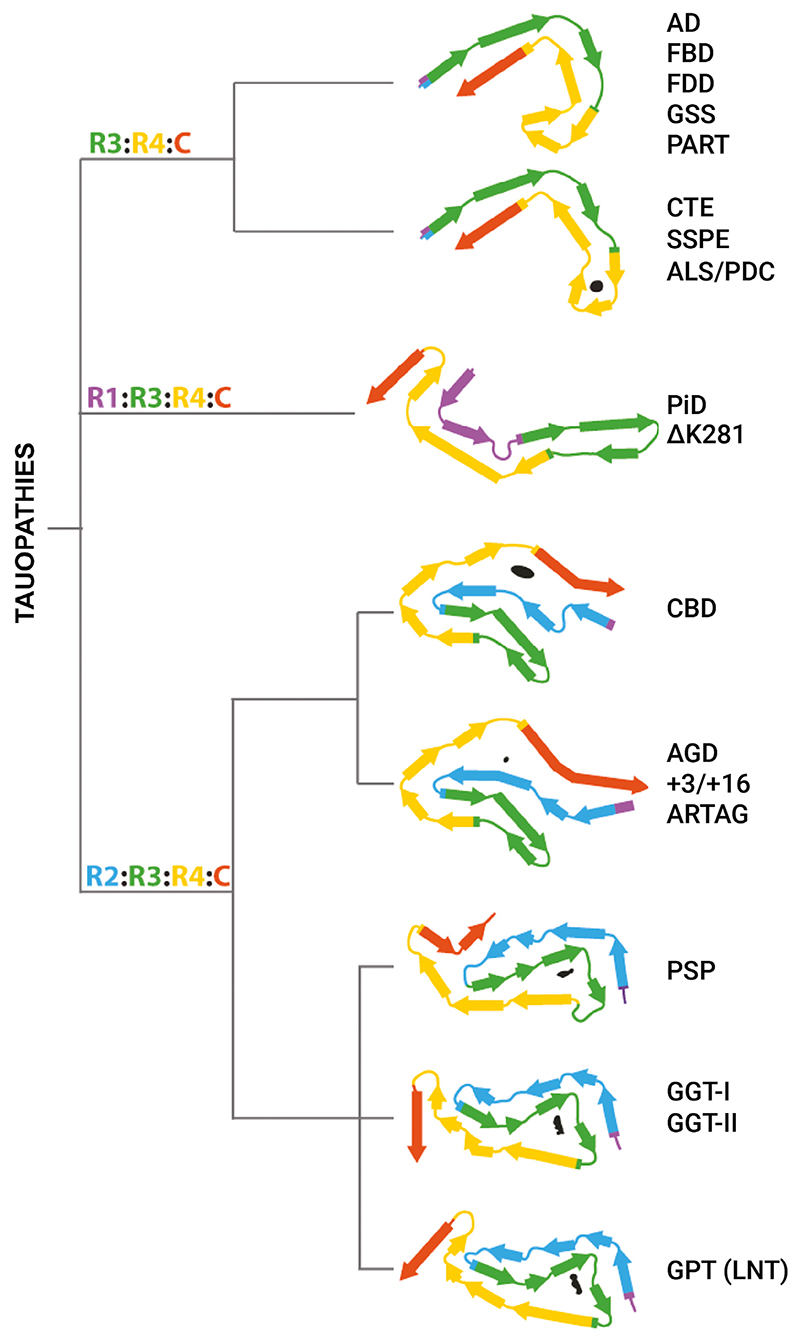
Structure-based classification of tauopathies. The dendrogram shows the proposed classification, with the corresponding folds displayed with the first β-strand in R3 oriented approximately horizontally, except for the globular glial tauopathy (GGT) and globular glial tauopathy-progressive supranuclear palsy-tau (GPT) folds, which are aligned to the progressive supranuclear palsy (PSP) fold. Internal, nonproteinaceous densities are shown in black. AD, Alzheimer’s disease; AGD, argyrophilic grain disease; ALS/PDC, amyotrophic lateral sclerosis/parkinsonism–dementia complex; ARTAG, age-related tau astrogliopathy; CBD, corticobasal degeneration; CTE, chronic traumatic encephalopathy; FBD, familial British dementia; FDD, familial Danish dementia; GSS, Gerstmann-Sträussler-Scheinker disease; LNT, limbic-predominant neuronal inclusion body 4R tauopathy; PART, primary age-related tauopathy; PiD, Pick’s disease; SSPE, subacute sclerosing panencephalitis.

In subsequent years, structures of tau filaments from PiD (Falcon, Zhang, Murzin, et al., 2018), CTE (Falcon et al., 2019), CBD (Arakhamia et al., 2020; Zhang et al., 2020), PART (Shi, Murzin, et al., 2021), AGD, PSP and GGT (Shi, Zhang, et al., 2021) were determined, giving rise to a structure-based classification of tauopathies (Figure 3; Shi, Zhang, et al., 2021). Tau filament structures from CTE brains are similar to those of AD, but differ by a larger cavity in the β-helix region and the presence there of a density of unknown identity. In PiD, the tau filament core consists of the C-terminal two thirds of R1, the whole of R3 and R4, and 10–13 amino acids after R4.

In CBD, AGD, PSP, and GGT, the cores of tau filaments consist of the whole of R2, R3, and R4, and 10–13 amino acids after R4. CBD and AGD folds are four layered, whereas PSP and GGT folds are three layered. We also identified a three layered fold intermediate between those of GGT and PSP in a case of atypical PSP. We suggested that this globular glial tauopathy-progressive supranuclear palsy-tau (GPT) fold gives rise to a new clinicopathological entity that we named limbic-predominant neuronal inclusion body 4R tauopathy (LNT) (Shi, Zhang, et al., 2021).

Specific tau folds characterize different diseases, but several conditions share a fold. FBD, FDD, PART, and cases of Gerstmann–Sträussler–Scheinker disease have the Alzheimer tau fold in common (Hallinan et al., 2021; Shi, Murzin, et al., 2021; Shi, Zhang, et al., 2021). The CTE tau fold is also found in subacute sclerosing panencephalitis (SSPE) (Qi, Hasegawa, et al., 2023) and the amyotrophic lateral sclerosis/parkinsonism–dementia complex (ALS/PDC) of the island of Guam and the Kii peninsula of Japan (Qi, Verheijen, et al., 2023). A familial form of PiD is characteristic of cases with mutation ΔK281 in *MAPT* (Schweighauser, Garringer, et al., 2023). Moreover, tau filaments from AGD, aging-related tau astrogliopathy, and cases of mutations in intron 10 of *MAPT* share a common fold (Shi, Zhang, et al., 2021). The structures of filaments from these cases with *MAPT* mutations indicate that the relative overproduction of wild-type 4R tau can give rise to the AGD fold, whereas that of wild-type 3R tau generates the Pick fold.

*Postmortem* human tau filament structures display additional densities. Many densities face outwards into the surrounding solvent or the fuzzy coat, whereas others are buried within the ordered filament cores. These densities (which may correspond to posttranslational modifications of tau or noncovalently bound cofactors) may play an important role in giving rise to a particular tau fold, but their identities remain to be discovered.

## 6 Conclusion

The assembly of amyloid filaments using purified recombinant proteins has been used extensively to study mechanisms underlying amyloid formation. Cryo-EM has yielded structures of in vitro assembled tau filaments (Abskharon et al., 2022; Zhang et al., 2019), but none were identical to those of filaments extracted from human brains. Tau filaments from transgenic mouse models also have a different appearance from those in human diseases (Schweighauser, Murzin, et al., 2023).

What is needed, then, are methods by which one can form tau filaments with structures like those from human brains. So far, this has only been possible for fragments of tau (Lövestam et al., 2022). In this system, N- and C-terminal truncations were shown to be critical for forming PHFs. The addition of 100–200 mM sodium chloride led to the formation of tau filaments with the CTE fold.

The ordered assembly of tau is believed to be the gain of toxic function that causes human tauopathies (reviewed in Goedert, 2016). Downstream, propagation of assembled tau and neurodegeneration take place. Short tau filaments are the major species responsible for propagation, at least in transgenic mice (Jackson et al., 2016). The tau species that lead to neurodegeneration remain to be identified. Mechanisms of propagation and neurodegeneration are probably linked and may be influenced by the structural differences between tau filaments that have been identified by cryo-EM (reviewed in Scheres et al., 2023). Elucidating these mechanisms will be key for developing safe and effective therapies for these diseases.
